# Evolving multispectral sensor configurations using genetic programming for estuary health monitoring

**DOI:** 10.1080/03036758.2024.2393297

**Published:** 2024-08-21

**Authors:** Mitchell Rogers, Mihailo Azhar, Stefano Schenone, Simon Thrush, Bing Xue, Mengjie Zhang, Patrice Delmas

**Affiliations:** a School of Computer Science, The University of Auckland, Auckland, New Zealand; b Institute of Marine Science, The University of Auckland, Auckland, New Zealand; c School of Engineering and Computer Science, Victoria University of Wellington, Wellington, New Zealand

**Keywords:** Hyperspectral imaging, genetic programming, wavelength selection, organic matter, porosity, sediment

## Abstract

Assessing ecosystem health on a large scale is crucial for a wide range of management and regulatory decisions. Technologies such as hyperspectral imaging allow noninvasive and rapid estimation of key attributes based on observed reflectance. However, these images are high-dimensional and real-world applications require models based on fewer wavelengths. This paper proposes a new wavelength selection and feature extraction method for hyperspectral image analysis based on genetic programming to automatically select key wavelength regions and informative image features. A dataset of hyperspectral images of sediment in the field was collected and paired with ground-truth measurements of the sediment porosity and organic matter content. Two new program structures were proposed to construct feature extraction trees from either the mean reflectance spectra (spectra-based) or full hyperspectral images (image-based). SVR models were constructed to predict attributes based on the extracted features. Various regression models were used to predict the porosity and organic matter content. Full-wavelength models were constructed to reliably predict the organic matter content. The proposed spectra-based genetic programming solutions show competitive results compared to common wavelength selection methods, such as SPA, CARS, and RC. Finally, the best-evolved solution was applied to predict sediment organic matter content across all collected images.

## Introduction

Assessing the health of benthic habitats and understanding ecosystem dynamics on a large scale is important for many regulatory and management decisions (Pettorelli et al. 2014; Senf 2022). Species in these ecosystems bioturbate and bioirrigate sediments, recycling nutrients such as nitrogen and carbon, thereby improving the quality of the water bodies they inhabit (Schenone et al. 2019). Traditional methods for assessing the health of marine areas use sparse sampling and extrapolated to a larger region (Martin et al. 2015). However, with the development of satellite and drone technology, along with more complex imaging methods including hyperspectral imaging, it is possible to map vast areas quickly and regularly (Finn et al. 2011; Pettorelli et al. 2014; Azhar et al. 2020; Senf 2022). Hyperspectral imaging captures the reflectance of a target area with hundreds of narrow wavelengths across the electromagnetic spectrum, and saves the result as a hyperspectral image. As with spectroscopy, which captures a single-point measurement, it is possible to relate spectral information to the chemical composition of the target.

Compared to spectroscopy, hyperspectral imaging considers the spatial arrangement of pixels, allowing texture features (also referred to as spatial or image features) to be used along with spectral features to predict the attributes of interest (Jia et al. 2019; Behera et al. 2021). However, a major drawback of hyperspectral images are their high dimensionality, which results in long capture and processing times (Cheng et al. 2017). In addition, images captured at adjacent wavelengths often include redundant information due to the highly correlated signals and many are uninformative for specific prediction tasks. Wavelength selection is a solution to this problem, where one can select the wavelengths that contain the most informative spectral information to help predict the attributes of interest, and then design a lower-cost multispectral sensor to predict these attributes in real time (Liu et al. 2014).

Existing wavelength selection methods ignore the correlation between adjacent wavelengths, treating wavelength selection as a combinatorial optimisation problem, and informative image features are often extracted from wavelengths selected for their informative spectral features (Rogers et al. 2023). A flexible wavelength selection method that can select variable-sized intervals to take advantage of the correlation between adjacent wavelengths is yet to be developed.

Hyperspectral imaging and spectroscopy have been applied to study the quality of soil for some time to predict moisture (Finn et al. 2011), nutrient content (Peng et al. 2021), and salinisation (Abd El-Hamid and Hong 2020). However, these studies were limited to laboratory measurements of soil attributes. In this study, we aim to utilise hyperspectral imaging to predict two attributes of sediment: organic matter content and porosity. Using land-based surface reflectance measurements, we will design a lower-cost multispectral model incorporating image features to predict these attributes of interest. While a study found that soil reflectance is influenced by organic matter content (Reis et al. 2021), the porosity of sediment is yet to be investigated using hyperspectral imaging.

The need for flexible, interpretable methods capable of dealing with high dimensionality and learning reliable models from small datasets has led to the development of a genetic programming (GP)-based approach. GP is an evolutionary computation method that can evolve programs represented by trees of functions to solve problems (Koza 1994; Banzhaf et al. 1998), thereby allowing us to search the space of valid function trees of varying complexity. The advantages of GP include better interpretability (Mei et al. 2023), and the ability to combine multiple steps in an image processing pipeline. Typically, GP does not require as many training samples as many popular deep-learning methods (Bi et al. 2019).

This study proposes two new GP-based approaches that incorporate multiple steps found in a typical hyperspectral image analysis process, including wavelength selection, image feature extraction, and modelling, to create simple and interpretable function trees representing a multispectral model. The two approaches, image-based and spectra-based, are compared to identify whether including image features improves the model performance. These two GP-based approaches incorporate application-specific functions that are inaccessible to other wavelength selection techniques, such as wavelength interval selection. We believe these features will give this algorithm an advantage over other wavelength selection methods. These approaches were applied to a novel dataset to predict the sediment porosity and organic matter content of benthic habitats based on surface reflectance data.

The main contributions of this study can be summarised as follows:

(1) A collection of hyperspectral images of sediment collected from an intertidal area along with corresponding ground-truth porosity and organic matter content measurements.
(2) Two new GP program structures: one to extract informative features from hyperspectral images and the other from the mean reflectance spectra. The function sets used by these program structures include interval selection, image filtering, and image-based functions to create explainable feature extraction trees for multispectral regression models.
(3) A comparison of the GP-based solutions to models based on widely adopted wavelength selection methods, and the full set of wavelengths.
(4) An investigation into the applicability of the best GP individual for predicting the organic matter content across multiple unseen hyperspectral images.

## Background and related work

This study incorporates elements of hyperspectral imaging, wavelength selection, and GP. The following section introduces the background of the above research areas and related works.

### Hyperspectral imaging

Hyperspectral imaging is an imaging technique that captures information across the electromagnetic spectrum and was initially developed for remote sensing applications in the 1980s (Goetz et al. 1985). Since then, it has been extensively used in agriculture and food science applications (Su and Sun 2018; Ma et al. 2019; Feng et al. 2021). Previous hyperspectral imaging studies investigating sediment and soil typically manually sampled the soil at the surface level and collected spectral measurements in controlled laboratory environments (Reis et al. 2021; Peng et al. 2021). Some of these studies aimed to build models to predict the same attributes using remote sensing data (Abd El-Hamid and Hong 2020; Peng et al. 2021). However, models built in controlled laboratory environments may not be sufficiently accurate when applied to surface reflectance images from outside laboratory environments.

Reducing the dimensionality of hyperspectral images through wavelength selection is an essential step in obtaining rapid and low-cost multispectral models (Wu and Sun 2013; Zhang et al. 2021). Current wavelength selection techniques treat wavelength selection as a combinatorial optimisation problem, where each wavelength is treated as an independent measurement. The full set of wavelengths consists of evenly distributed measurements of the continuous electromagnetic spectrum, each covering a couple of nanometres, where the native spectral resolution of the sensor constrains the width of each wavelength. However, aggregating or binning adjacent wavelengths can yield broader and potentially less noisy spectral features (Finn et al. 2011). In addition, incorporating narrow wavelengths into custom multispectral sensors is more expensive because they require narrower bandpass filters.

The spatial information contained in hyperspectral images has been shown to be useful in several studies. NIR (900–1700 nm) hyperspectral imaging has been used to classify soil into different texture groups (e.g. clay, loam, and sand) (Jia et al. 2019) and predict the total nitrogen content of a range of soil types (Behera et al. 2021). These studies extracted four grey-level co-occurrence matrix (GLCM) texture descriptors: contrast, correlation, energy, and homogeneity, averaging each descriptor over four angles. Both studies, including these image texture features, resulted in models with better performance in predicting the soil attributes. Image features were typically extracted from wavelengths deemed informative based on spectral features. This approach assumes that the most informative image features are located at the same wavelengths as the most informative spectral features.

Hyperspectral imaging is often limited to small datasets because large hyperspectral imaging datasets are costly and time-consuming to collect owing to the low sampling rate and cost of laboratory analysis (Zhou et al. 2019; Hennessy et al. 2020; Khan et al. 2021). In addition, explainable solutions are preferred to correlate the selected wavelengths with known chemical absorption bands. For example, the spectral responses of micro- and macro-organisms can be discriminated between and have been associated with the absorption bands of molecules such as chlorophyll and carotenoids (Montes-Herrera et al. 2021). In this paper, we aim to develop a new interpretable wavelength selection method that can select variable-width wavelength intervals, select wavelengths with informative image features, and apply this to a new dataset of sediment images collected in situ from the estuary surface.

### Genetic programming (GP)

This need for explainability, coupled with the constraint of small datasets, naturally leads to the adoption of GP, which is well suited to these conditions. GP represents solutions as program trees and conducts a search for solutions in the space of all valid program trees (Koza 1992; Banzhaf et al. 1998). Representing solutions as computer programs allows multiple stages of the processing pipeline to evolve simultaneously, such as filtering and feature extraction operations traditionally used in computer vision tasks (Shao et al. 2014; Bi et al. 2021). Designing multispectral sensors requires expertise from many disciplines, such as domain experts from the application area, computer programmers, and engineers (Su and Sun 2018). By incorporating multiple steps that require domain expertise into GP function trees, GP applications in hyperspectral imaging may be able to reduce the need for expert knowledge to design these pipelines.

GP has been successful in many image-related tasks, such as feature learning for image classification (Bi et al. 2019; Fan et al. 2023) and image segmentation (Vojodi et al. 2013; Liang et al. 2017), with most of the previous methods focussing on RGB or grayscale images. GP solutions are inherently interpretable because their tree-based structure comprises simple functions and has shown good performance compared with other learning methods (Bi et al. 2021).

Over the past few decades, there have been multiple applications of GP to hyperspectral, multispectral, and spectroscopy data. Some of these methods use range or interval operators to exploit the continuous spectral data (Kaleita et al. 2006; Chion et al. 2008) or image features (Momm et al. 2009; Dozal et al. 2018). However, few methods have considered both image features and functions specific to spectral data, and even fewer have used GP to regress attributes from hyperspectral images.

## Materials and Methods

This section outlines the data collection and processing methods, along with the details of the proposed algorithm, comparison methods, and experiments.

### Data collection

**Figure 1.**
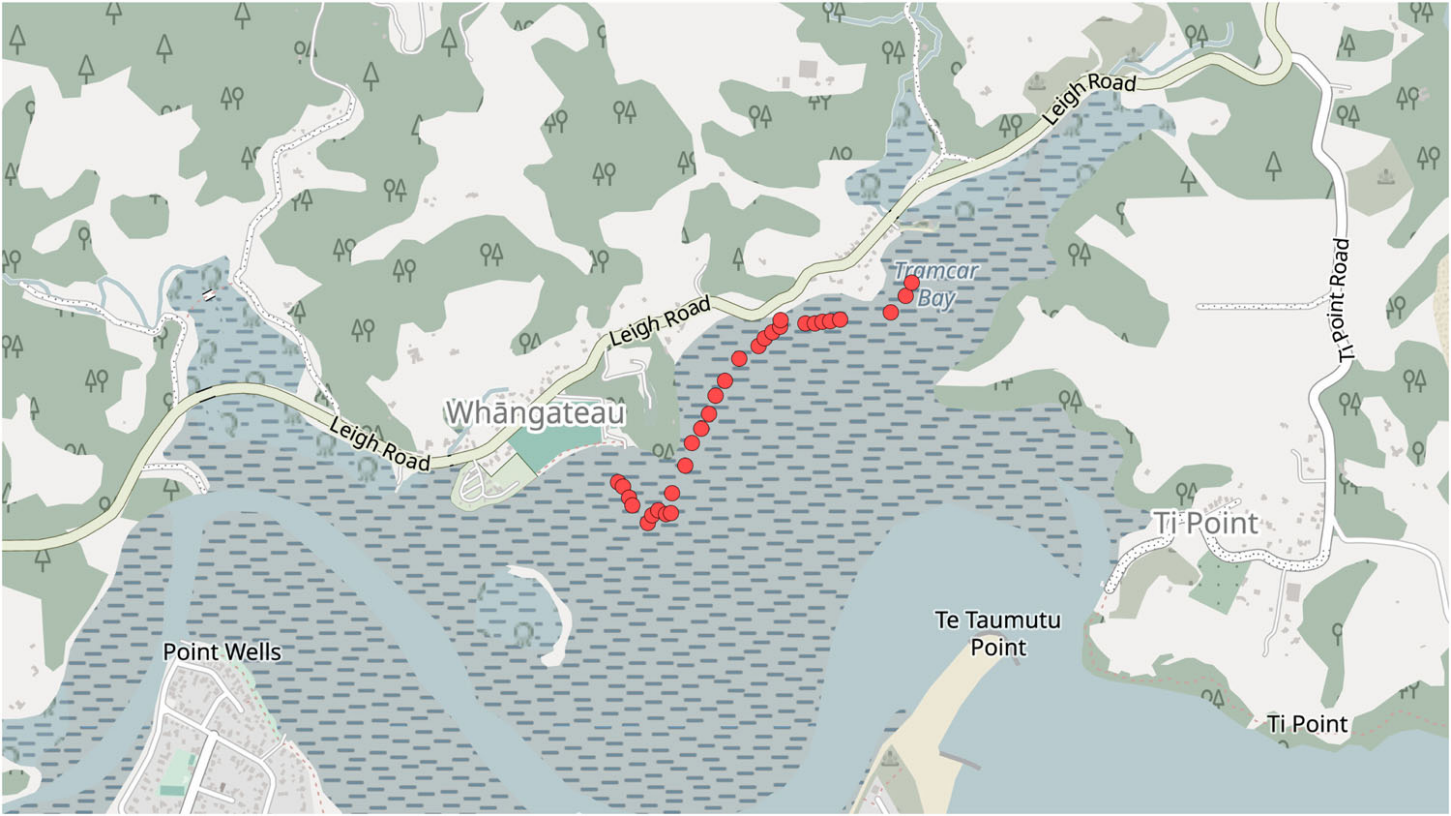
Locations of the 30 sample points in the Whangateau Harbour.

Sediment samples and hyperspectral images were acquired on the 13th of July, 2022 between 11:50 am and 3:10 pm, to coincide with low tide and peak sunlight in Whangateau Harbour, New Zealand. Quadrats (25×25cm) were placed on the sediment surface at 30 locations (Figure 1). Quadrats were located to avoid submerged sediment, regions with many shells, and human footprints.

**Figure 2.**
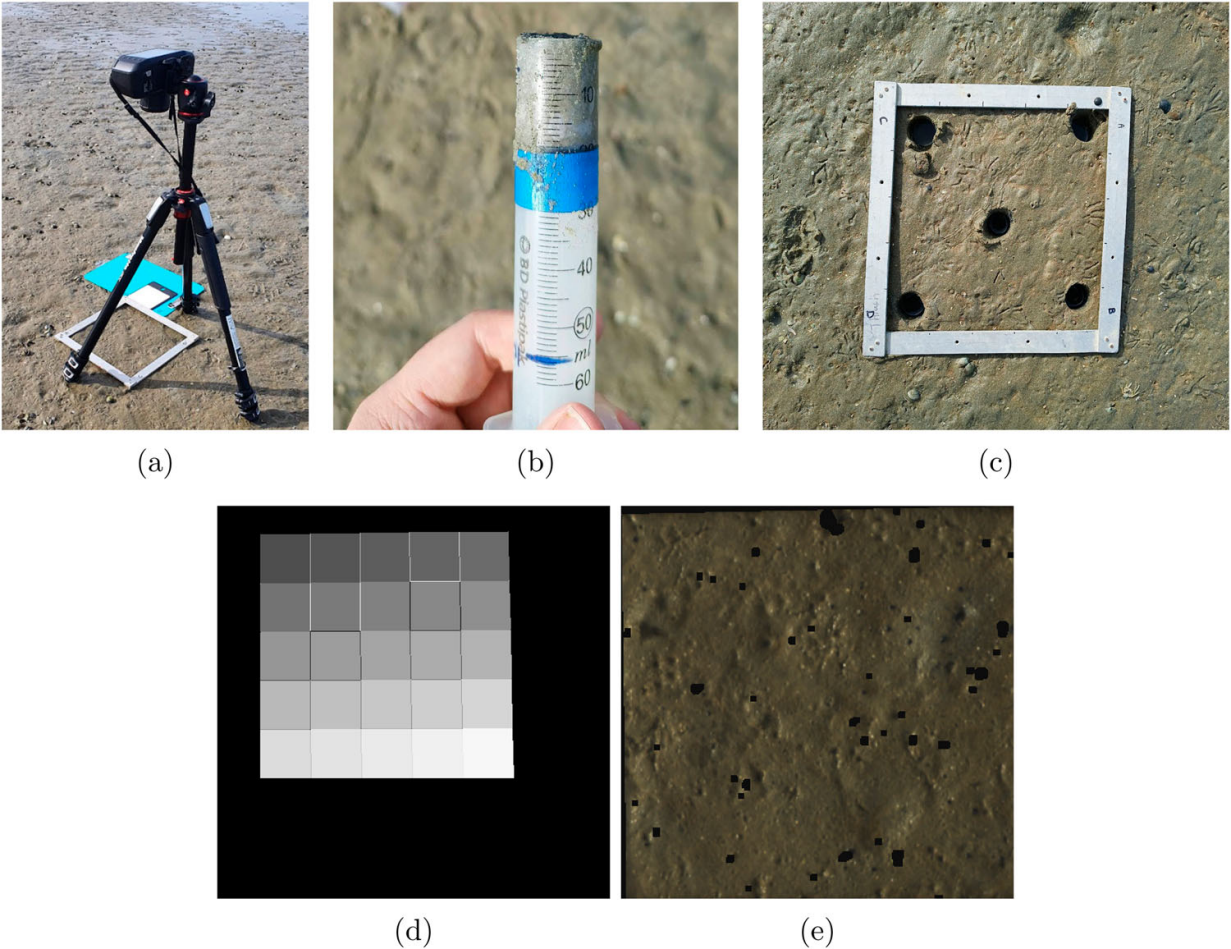
Data collection, annotation and ROI subsampling process. **A,** Camera and tripod over the quadrat area. **B,** Sediment sample extraction syringe. **C,** Sampling areas of the quadrat. **D,** Visualisation of the 5 by 5 regions of interest within the quadrat. **E,** RGB representation of the greater ROI, after calibration, and background and shell segmentation.

Hyperspectral images were acquired using a portable SPECIM IQ camera (Spectral Imaging Ltd., Oulu, Finland), capturing 204 bands within a wavelength range of 397.32–1003.58 nm at a spectral resolution of 7 nm (FWHM) and an image size of 512×512 pixels. The portability of this hyperspectral camera facilitates the gathering of data in natural environments, thereby streamlining field research (Behmann et al. 2018). During the image acquisition, the camera was fixed on a tripod 70 cm above the surface (Figure 2A). The spatial resolution at this height was approximately 0.75 mm per pixel. A Teflon white reference tile (approximately 99% reflectance) was placed in the camera view next to the quadrat for reflectance calibration. The camera automatically obtained a dark reference for each image by covering the sensor and capturing the background signal. Due to the intermittent presence of clouds, the exposure time varied from 5 to 7 ms.

Each quadrat was divided into five evenly spaced rows and columns (5 cm each) to sample sediment from the four corners and the direct centre (row 3, column 3) of the quadrat (as shown in Figure 2C). Five sediment samples were collected from each quadrat using a syringe core to a depth of 3 cm (core volume $18.5\;{\mathrm{cm}}^{3}$) (Danovaro 2009) (Figure 2B). Each core was stored in an individual container and placed in an icebox to prevent liquid evaporation and bacterial organic matter remineralisation. In total, 150 sediment samples were collected. If large shells or rocks obstructed the sample core, the sample was discarded and the ground truth sample was taken from the adjacent position. Upon returning from the field, sample containers were stored in a freezer ($-20^{\circ }C$) until subsequent processing.

### Image preprocessing

Because of the image overexposure in the visible spectrum, some white reference values were outside the maximum value captured by the sensor. A correction operation was applied to estimate the overexposed values by optimising the transformation required to fit the non-overexposed values to a seed white reference curve ($R_{\mathrm{seed}}$). As shown in Equation (1), the values for *a*, *b*, and *c* were optimised to minimise the difference between the know values ($R_{\mathrm{initial}}$) and the corresponding seed spectrum values ($R_{\mathrm{seed}}$) for each wavelength *i*.

(1) $$R_{\mathrm{corrected}}[i]=\left\{ \begin{array}{ll} a\times R_{\mathrm{seed}}[i{]}^{b}+c, & \text{ if }R_{\mathrm{initial}}[i]>\mathrm{Maximum}\;\mathrm{sensor}\;\mathrm{value}\\ R_{\mathrm{initial}}[i], & \text{ otherwise } \end{array}\right.$$

Finally, each image was automatically rescaled using the corrected white reference curve and dark reference. Pixel values of each wave band were rescaled using the normal reflectance calibration method where $R_{c}=(R_{R}-R_{D})/(R_{w}-R_{D})$, where $R_{c}$ is the corrected reflectance value, $R_{R}$ is the raw reflectance, $R_{W}$ is the white reference, and $R_{D}$ is the dark reference value.

The area inside each quadrat was divided into a five-by-five grid (Figure 2D). Each cell in the grid was saved as an individual subimage. In addition to these observations, an ROI mask was saved by delimiting the portion of the image that contained relevant pixel values. Five of the 25 subimages were assigned to a ground-truth reference measurement. The remaining 20 samples were collected to visualise the modelling results. The pixel values within the images were standardised using the standard normal variate (SNV) (Barnes et al. 1989) approach to reduce the effect of reflection intensity variations across the image, and the individual pixels were smoothed using a Savitzky-Golay filter (Savitzky and Golay 1964). Shells and other bright highlights in the image were automatically segmented using a threshold wavelength of 873 nm, where the shells exhibited a high spectral response compared to the low response of the sediment, followed by morphological erosion. Figure 2E shows an example of a quadrat after processing and removing specular components. Finally, the pixel values of each region of interest were averaged to obtain the mean reflectance spectra.

### Reference measurements

Sediment samples were analysed for porosity and organic matter content. Porosity was measured as the ratio of interstitial volume to total sediment volume (Danovaro 2009). The organic matter content was measured as loss on ignition at $450^{\circ }C$ (Parker 1983). One sediment sample for the measurement of organic matter content was removed because of an error during laboratory analysis.

The reference samples were split into calibration and prediction sets at a ratio of 2:1, using the sample set partitioning based on joint x-y distances (SPXY) method (Galvão et al. 2005). The SPXY algorithm maximises the variation in both sets' dependent (reference measurements) and independent variables (mean reflectance spectra) by selecting samples with the highest minimum Euclidean distance from previously selected samples. Table 1 describes the summary statistics for porosity and organic matter. The dataset is available on FigShare (see the data availability statement).

**Table 1.** Details of the dataset splits for each of the attributes of interest.

Attribute	Split	No. Samples	Mean	Std dev	Min	Max
Porosity	Calibration	100	40.32	4.05	26.92	54.61
	Prediction	50	40.17	2.35	35.53	47.88
Organic matter content	Calibration	100	1.1	0.35	0.52	2.09
	Prediction	49	1.08	0.27	0.52	1.85

### Algorithm overview

This study proposes two GP program structures to generate individuals: one taking hyperspectral images as inputs (image-based) and another taking mean reflectance spectra (spectra-based). Initial candidate GP trees (individuals) were generated by combining the functions and terminals, according to the program structure criteria. Each individual received either a hyperspectral image or a mean spectral vector as the input, and the result was a feature vector used to train an SVR model. SVR models were used because they can be fitted efficiently, but the extracted features can be used with other modelling methods after the trees have evolved.

At the beginning of the evolutionary process for each run, the calibration set was randomly split into training and evaluation portions in a 70:30 ratio. Feature vectors were extracted from all training and evaluation samples, and the extracted features were standardised across all extracted feature vectors (based on Equation (2)). The SVR models were then fitted using the training portion, and performance was evaluated using the evaluation portion, which provided the fitness of each individual. The split of the calibration set varied between each run; however, the test set remained completely independent and was not considered for the fitness evaluation. Two fitness functions are compared: $R^{2}$ (Equation (3)) and the mean squared error (MSE) (Equation (4)) values of the models applied to the evaluation portion of the calibration set.

(2) $$X_{s}=\frac{X-\bar{X}}{\sigma_{X}}$$

(3) $$R^{2}=1-\frac{\sum_{i}(y_{i}-\hat{y_{i}}{)}^{2}}{\sum_{i}(y_{i}-\bar{y}{)}^{2}}$$

(4) $$\mathrm{MSE}=\frac{1}{n}\sum_{i=1}^{n}(y_{i}-{\hat{y}}_{i}{)}^{2}$$

**Figure 3.**
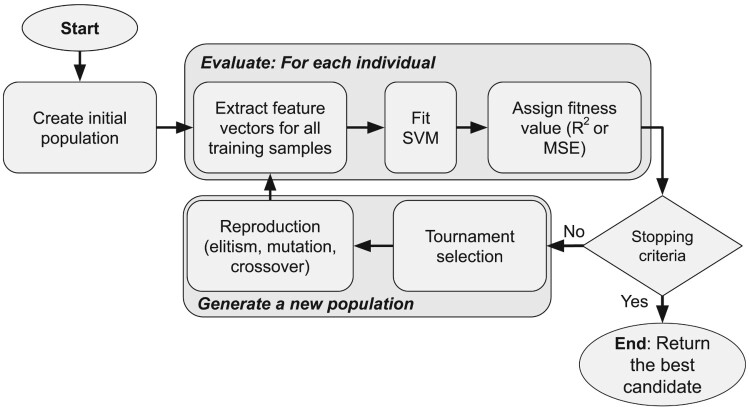
Evolutionary algorithm steps.

The evolutionary process continues by evaluating the fitness of each individual, selecting individuals with tournament selection, and updating the population using *elitism*, *crossover*, and *mutation* operators. The evolutionary process is repeated for a fixed number of generations. Finally, the algorithm returned the fittest individual from the previous generation as the final solution. The steps of this process are summarised in Figure 3.

### Program structure

**Figure 4.**
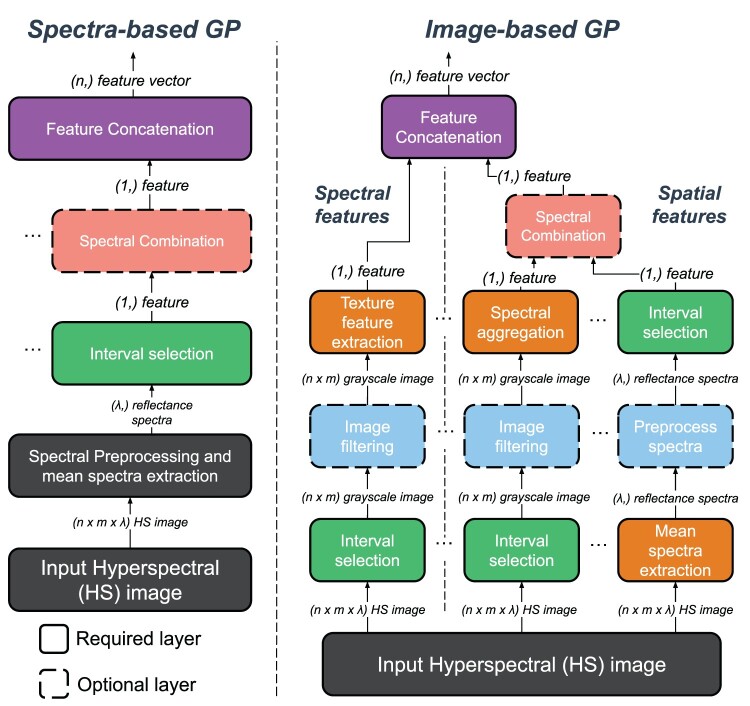
The two program structures of the STGP individuals. The left side shows the structure of the individuals designed to process the mean spectral responses and the right side shows the structure for processing hyperspectral images. Both implementations produce a variable-length feature vector by considering a variable number of feature extraction branches as inputs to the feature concatenation layer. For example, the right-side implementation concatenates one spectral feature and one texture feature to produce a feature vector of length two.

Figure 4 shows the two proposed program structures, where the left side shows the program structure of the mean spectral response, and the right side shows the program structure for processing hyperspectral images. A typical pipeline for hyperspectral image analysis first averages the spectral values over a representative region of interest, optionally applies spectral pretreatment, selects informative wavelengths, and then fits a model to these informative wavelengths (Cheng et al. 2017; Ma et al. 2019; Özdoğan et al. 2021). This process has a significant impact on the design of both program structures, allowing individuals to extract informative spectral characteristics by selecting the optimal wavelengths from hyperspectral images.

The spectral-based structure takes an input preprocessed reflectance spectrum, selects an important wavelength, and optionally applies arithmetical operations to multiple selected wavelengths, forming a single spectral feature extraction branch. Finally, the feature concatenation step combines a variable number of feature-extraction branches into a single feature vector.

The image-based program structure involves two feature extraction sequences to extract image and spectral features independently. This structure extends the spectral-based version to allow for the selection of spectral and spatial information in a manner similar to traditional computer vision methods. In addition to the technique utilised in the spectral-based structure, a spectral feature may be created by selecting a single-channel wavelength image from the hyperspectral image, optionally applying various image filters, and computing the average value across the region of interest. A texture descriptor can replace averaging the region of interest to produce an image feature. Finally, the features from the variable number of feature extraction branches are concatenated into a single feature vector.

These structures allow a variable number of feature-extraction sequences to produce variable-sized feature vectors. For example, an optimal solution may consist entirely of or have no spectral features. The evolutionary process determines the adequate number of branches and the optimal number of each branch type for a specific problem.

### Function set

The program structures contain five types of functions: interval selection, filtering/preprocessing, spectral combination, aggregation, and concatenation. The spectral-based method uses a smaller function set, which is a subset of the image-based method function set, applying functions to the mean reflectance spectra rather than to full hyperspectral images. Table 2 describes the complete function set along with the corresponding evolved parameters and the input and output types.

**Table 2.** Set of available functions. The set column designates whether the terminal is used by the spectra- or image-based GP structures.

Function name	Input types	Output type	Set
Interval selection			
Image interval selection (Mean)	HSI, *w*, *k*	GSI	Image-based
Image interval selection (Gaussian)	HSI, w,λ,k,σ	GSI	Image-based
Image interval selection (Median)	HSI, w,λ,k	GSI	Image-based
Spectra interval selection (Mean)	Spectra, w,λ	SF	Both
Spectra interval selection (Median)	Spectra, w,λ	SF	Both
Image filtering and normalisation			
Gaussian filter	GSI, k,σ	GSI	Image-based
Derivative of Gaussian	GSI, *σ*	GSI	Image-based
Median filter	GSI, *k*	GSI	Image-based
Gabor filter (7×7)	GSI, f,θ	GSI	Image-based
Gabor filter (9×9)	GSI, f,θ	GSI	Image-based
Max filter	GSI, *k*	GSI	Image-based
Min filter	GSI, *k*	GSI	Image-based
Min-max scaling	GSI	GSI	Image-based
Spectral preprocessing			
Savitzky-Golay filtering	Spectra	Spectra	Image-based
Standard Normal Variate (SNV)	Spectra	Spectra	Image-based
Aggregation			
Spectral aggregation	GSI	SF	Image-based
Mean spectra extraction	HSI	Spectra	Image-based
Image features			
GLCM feature	GSI, $d,\theta_{g}$	FV	Image-based
Gray-level histogram feature	GSI	FV	Image-based
Spectral combination			
Arithmetic operations (+, −, ×, ÷)	2 SFs	SF	Both
Convert to feature vector type (=)	SF	FV	Both
Feature concatenation			
Root 2	2 FVs	FV	Both
Root 3	3 FVs	FV	Both
Root 4	4 FVs	FV	Both

Note: The acronyms HSI, GSI, SF, and FV refer to the types: hyperspectral image, grayscale image, spectral feature, and feature vector, respectively.

Interval selection methods extract spectral features and waveband images over variable-size intervals. Evolved individuals can select informative intervals from the full hyperspectral image or mean reflectance spectrum. The function set includes three per-pixel averaging techniques (mean, median, and Gaussian) to extract grayscale images from a hyperspectral image over a window of selected wavelengths. Each interval selection method requires a central wavelength and a window size. When a portion of the window was outside the spectral range of the data, the average was calculated only for the section within that range. The averaging weights were normalised to the sum of one.

After extracting a grayscale image, GP individuals can implement optional filtering and normalisation operations. The available operations include standard filters such as Gaussian, the derivative of Gaussian, median, min, and max filters with variable kernel sizes, and Gabor filters with two window sizes. For normalisation, the feature set includes min-max normalisation.

This function set includes both spectral and image features. Spectral features can be extracted by averaging pixel intensities over a grayscale image (spectral aggregation) or by selecting an interval from the mean spectra. The mean spectra extraction operation provides the mean intensity of each band over the defined region of interest (ROI). The following optional step applies standard spectral pretreatment techniques to preprocess the spectrum: Savitzky-Golay filtering (Savitzky and Golay 1964) or SNV. The individual then selects an interval and averages the spectral response vector over a window centred at a selected wavelength.

The extracted spectral features can then be combined into indices using basic arithmetic operations, before converting them into a single feature. Spectral features are generated either through interval selection applied to the extracted reflectance spectra, or from spectral aggregation (averaging) over a grayscale image. Finally, individuals concatenate the extracted set of spectral and image features to form a single feature vector of length *n* using root operations.

This function set includes three image feature types: Gabor filters, GLCM features, and histogram statistics. These three function sets were selected because they are the most commonly used image texture descriptors in previous hyperspectral imaging research with wavelength selection (Rogers et al. 2023).

#### Gabor filters

Gabor filters apply a convolution with a filter created using Gaussian and sinusoidal terms (Fogel and Sagi 1989). Previous studies have used Gabor filters to predict intramuscular fat (Kucha et al. 2021) and total volatile base nitrogen (TVB-N) content in pork (Guo et al. 2018). Guo et al. (2018) used Gabor filters with a fixed standard deviation (σ=1) and frequency (*f* = 16) parameter but varied the angle over four directions (θ=0, π/4, π/2, and 3π/4), averaging over the four directions to produce a single rotationally invariant feature. Kucha et al. (2021) applied the same methodology to extract Gabor features over four angles with a fixed frequency and standard deviation. The function set includes two sizes of Gabor filters, 7×7 and 9×9 pixels, and allows the evolutionary process to select the orientation and frequency of the filters using random constants. The standard deviation (σ=2), aspect ratio (γ=0.3), and phase offset (ψ=1) are fixed.

#### GLCM features

GLCM features (Haralick et al. 1973; Haralick 1979) are calculated from two-dimensional matrices, where each entry (p(i,j)) counts the number of co-occurrences of a pair of pixel intensities (*iandj*) separated by a fixed distance (*d*) and angle ($\theta_{g}$). Feature descriptors describe the global distribution of pixel intensities and provide a single feature for describing the entire image. Haralick et al. (1973) and Haralick (1979) defined a set of 14 features to describe GLCM matrices, and further work such as that by Soh and Tsatsoulis (1999), and Clausi (2002) extended the set of features further. The set of GLCM features in this function set was limited to five features: homogeneity (Equation (5)), entropy (Equation (6)), contrast (Equation (7)), correlation (Equation (8)), and energy (Equation (9)) based on the matrices generated with a pixel distance and an angle parameter.

Homogeneity describes the closeness of the intensity distributions to the diagonal values. A region of interest will have a high homogeneity value when the neighbouring pixels have a similar intensity.

(5) $$\mathrm{Homogeneity}=\sum_{i,j}\frac{\;p_{i,j}}{1+(i-j{)}^{2}}$$

Entropy measures the randomness in the image texture. The maximum entropy value is obtained when all possible pairs of pixel intensities share the same co-occurrence counts. A low entropy occurs when only a few pairs of pixel intensities exist.

(6) $$\mathrm{Entropy}=-\sum_{i,j}p_{i,j}\log_{2}p_{i,j}$$

Contrast measures the local variations in the GLCM, and dissimilar neighbouring pairs, such as alternating light and dark intensities, will have a higher score.

(7) $$\mathrm{Contrast}=\sum_{i,j}p_{i,j}(i-j{)}^{2}$$

Correlation measures the joint probability of the occurrence of pixel–intensity pairs. The correlation measure is high when there is a predictable relationship between neighbouring pixels, such as a gradient following the path of the angle or a single-colour image.

(8) $$\begin{array}{cc} \mathrm{Correlation} & =\sum_{i,j}\frac{(i-\mu_{i})(j-\mu_{j})p_{i,j}}{\sigma_{i}\sigma_{j}}\\ \mathrm{where}\; & \mu_{i}=\sum_{i,j=0}(i\cdot p_{i,j})\;\;\\ & \mu_{j}=\sum_{i,j=0}(j\cdot p_{i,j})\;\;\\ & \sigma_{i}=\sqrt{\sum_{i,j=0}p_{i,j}(i-\mu_{i}{)}^{2}}\\ & \sigma_{j}=\sqrt{\sum_{i,j=0}p_{i,j}(j-\mu_{j}{)}^{2}} \end{array}$$

The angular second moment (ASM) is the sum of the squared elements in the GLCM, also known as the uniformity. Energy is the square root of the ASM and measures the regularity of the image texture. An irregular texture with a high entropy has a small energy value.

(9) $$\mathrm{Energy}=\sqrt{\sum_{i,j}p_{i,j}^{2}}$$

#### Histogram statistics

The set of histogram statistics was limited to mean, standard deviation, skewness, and kurtosis. When extracted from an image, the mean feature becomes the mean spectral intensity, and when extracted from a Gabor-filtered image, the feature describes the mean Gabor intensity.

### Terminal set

The The terminals and their value ranges are shown in Table 3. The central wavelength (*λ*) defines the centre wavelength of the interval window for wavelength selection, and the window-width parameter defines the size of the window centred on the central wavelength. The sigma (*σ*) parameter represents the variation around the mean value of the Gaussian operator. Each filtering operation uses a kernel size parameter (*k*) to define the width and height of the filter kernel. The two Gabor filter functions take two arguments that determine the orientation (*θ*) and frequency (*f*) of the Gabor kernel. GLCM features take two parameters: the angle ($\theta_{g}$) and distance (*d*) between co-occurrences. The orientation parameter of the Gabor filter is limited to eight rotations of $45^{\circ }$, but only four for GLCM features, because opposite angles produce equivalent results. Each terminal value was randomly initialised based on the ranges listed in Table 3 and evolved during the evolutionary process.

**Table 3.** Set of available terminal values for arguments in functions. The set column indicates whether the terminal is used by the spectra or image-based GP structures.

Terminal	Symbol	Range of values	Set
Central wavelength band	*λ*	$\left[0,N_{w}-1\right]$	Both
Window width	*w*	{1,3,5,7,9,11}	Both
Sigma	*σ*	(0,5]∩R	Both
Kernel size	*k*	{3,5,7,9,11}	Image-based
Orientation of Gabor filter	*θ*	$[0,7]\cap Q\times \frac{\pi }{4}$	Image-based
Frequency of Gabor filter	*f*	$\pi \times 2^{-(1+0.5v)}$, where v∈{0,1,2,3}	Image-based
GLCM matrix angle	$\theta_{g}$	$\{0,1,2,3\}\times \frac{\pi }{4}$	Image-based
GLCM matrix distance	*d*	{1,2,3,4,5}	Image-based

### Initialisation

The *ramped half-and-half* method is the most popular method for generating the initial individuals. This method randomly selects either the *full* or *grow* method to generate an individual. The *full* generation method generates a tree where each terminal node is at the same randomly selected depth between the minimum and maximum initial depths. The grow generation method generates a tree in which each terminal node is at a depth between the minimum and randomly selected maximum depths (i.e. between the minimum and maximum initial depths).

The high arity of functions and the placement of functions that take others as arguments in the penultimate layer hinder the tree initialisation functions. Many primitives in the function set have an arity of two or more. Without a height constraint, this causes a massive increase in tree size.

An individual height limit was set for each function output type to improve the initialisation algorithm and mitigate the aforementioned problems. Upon reaching this height limit, the initialisation function selects a function with a different output type. This method transitions the branch towards the terminal nodes within the height constraints. The *grow* and *full* tree generation methods integrate this approach to prevent the branch depths from exceeding the maximum depth. For the *full* generation method, the pre-terminal nodes (i.e. primitives that use the hyperspectral images or reflectance spectra as inputs) were selected only when primitivedepth=targetdepth−1 to maintain the fullness of the tree.

### Comparison methods

The pixel spectra of the reference regions of interest were averaged into a single mean reflectance spectrum per sample for analysis using partial least-squares regression (PLSR), support vector regression (SVR) with linear and radial basis function (RBF) kernels, XGBoost (Chen and Guestrin 2016) and LightGBM (Ke et al. 2017) models. The mean reflectance spectrum was treated as the independent variable (X) and the organic matter content and porosity were treated as dependent variables (Y). The optimal number of latent variables (LVs) for the PLSR model was determined by minimising the root-mean-square error of cross-validation (RMSECV). The RMSECV was calculated using five-fold cross-validation with two repeats. The hyperparameters of the SVR models were optimised using a grid search. The training (calibration) set was randomly split into five segments, and each model, based on the full set of wavelengths, selected wavelengths, and features extracted by the GP individuals, was fitted to each combination of four segments and evaluated on the fifth segment and test set. The performance metrics for the training and testing portions of cross-validation and the test set were obtained by averaging the $R^{2}$ and MSE results across the five folds. XGBoost (Chen and Guestrin 2016) and LightGBM (Ke et al. 2017) are based on gradient boosting, which is used to train multiple weak decision trees to form a strong regression model. The parameters for these models were manually optimised for each attribute to reduce overfitting. Neural-network-based approaches were not used because of the risk of overfitting to the few samples in this dataset.

Four wavelength selection methods were applied to select influential spectral features for comparison: the successive projections algorithm (SPA) (Araújo et al. 2001), competitive adaptive reweighted sampling (CARS) (Li et al. 2009), and regression coefficients (RC) (Mehmood et al. 2012). SPA is the most widely used wavelength selection approach for creating multispectral models. SPA is a forward selection method that selects wavelengths using projection operations to minimise collinearity (Galvão et al. 2008). The CARS algorithm selects wavelengths based on the Darwinian principle of ‘*survival of the fittest*’ and Monte Carlo random sampling. The absolute regression coefficient weights determined the selection probability of each wavelength during random sampling and the best model was determined using the root mean squared error from cross-validation (RMSECV). RC is a filter approach in which wavelengths are ranked in order based on their importance for regression and thresholding eliminates features below a specific score. Wavelengths were selected using the non-maximum suppression of the absolute values of the coefficients. The full-wavelength and wavelength selection models were compared using the same metrics.

### Experiments

Table 4 lists the parameters of each GP program structure. These parameters were based on settings commonly used in the GP community, and considered the computational complexity of dealing with high-dimensional hyperspectral images. The population sizes were 2048 for both the spectra-based GP and image-based GP methods. The image-based method has a much higher computational cost, leading to a longer runtime.

**Table 4.** Parameter settings.

Parameter	Spectra-based GP value	Image-based GP value
Generations	50	50
Population size	2048	2048
Population generation	Ramped half-and-half	
Selection method	Tournament (size *k*=4)	
Crossover rate	0.8	0.8
Mutation rate	0.2	0.2
Elitism rate	0.01	0.01
Initialisation tree depth	3–6	4–7
Maximum tree depth	8	9

The high arity of primitives, particularly in the penultimate layer, results in most nodes being leaf nodes. Only non-leaf nodes were selected as valid crossover points to increase the variation in the crossover operator. The chosen mutation node was a non-leaf node with a probability of 75%, and a leaf node with a probability of 25%.

Trees were initialised to a depth of between 3–6 and 4–7 for the spectra and image-based variants, respectively. The initial minimum depth was determined to be the depth required to generate a valid tree. A maximum tree depth of eight and nine was imposed to limit the size of individuals for the spectra-based and image-based implementations, respectively. A higher tree depth is used for the image-based model to compensate for the image-based program structure requiring at least one additional processing step than the spectra-based method, specifically extracting the texture or spectral feature.

The functions of both program structures were implemented using OpenCV (Bradski 2000) functions except for min-max scaling, which was implemented with Numpy (Harris et al. 2020), and the median filter implemented in SciPy (Virtanen et al. 2020). Both program structures were implemented in Python based on the *DEAP* (Distributed Evolutionary Algorithms in Python) package (Fortin et al. 2012). The code for all experiments will be made available on GitHub^1^.

The mean reflectance spectra and ROI masks were precomputed and saved within the data structure of the hyperspectral images. Pre-computing the reflectance spectra eliminates the need to repeatedly calculate the reflectance spectra and reduces the computational burden. The selected grayscale feature images also inherited the ROI mask, allowing texture feature extraction from the ROI.

To determine the best setup for this model, we compare the two fitness functions of the spectral and image-based models. The GP models were run 50 times for each comparison to deal with random variations. We also compare the best models from each run with other common wavelength selection methods for hyperspectral image analysis. We show that the GP feature extraction trees can be effective with other regression models by fitting PLSR models to the extracted features.

## Results and discussion

This section analyses the hyperspectral images collected and compares the performance of the two GP-based methods to models based on the full set of wavelengths and three common wavelength selection methods. Additionally, this section investigates the wavelengths selected by each method and the performance of GP-based methods over the training period and investigates the applicability of the models to the full set of images.

### Spectral profiles

**Figure 5.**
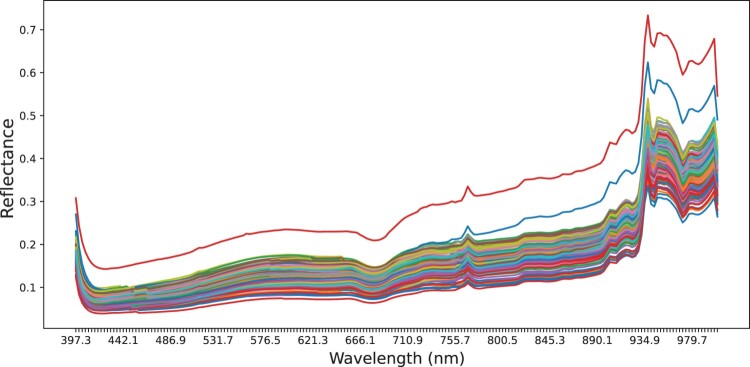
Mean reflectance of the 150 quadrat subregions. Variation above the 900 nm range is likely due to intermittent cloud cover and differences in water saturation of the sediment.

**Figure 6.**
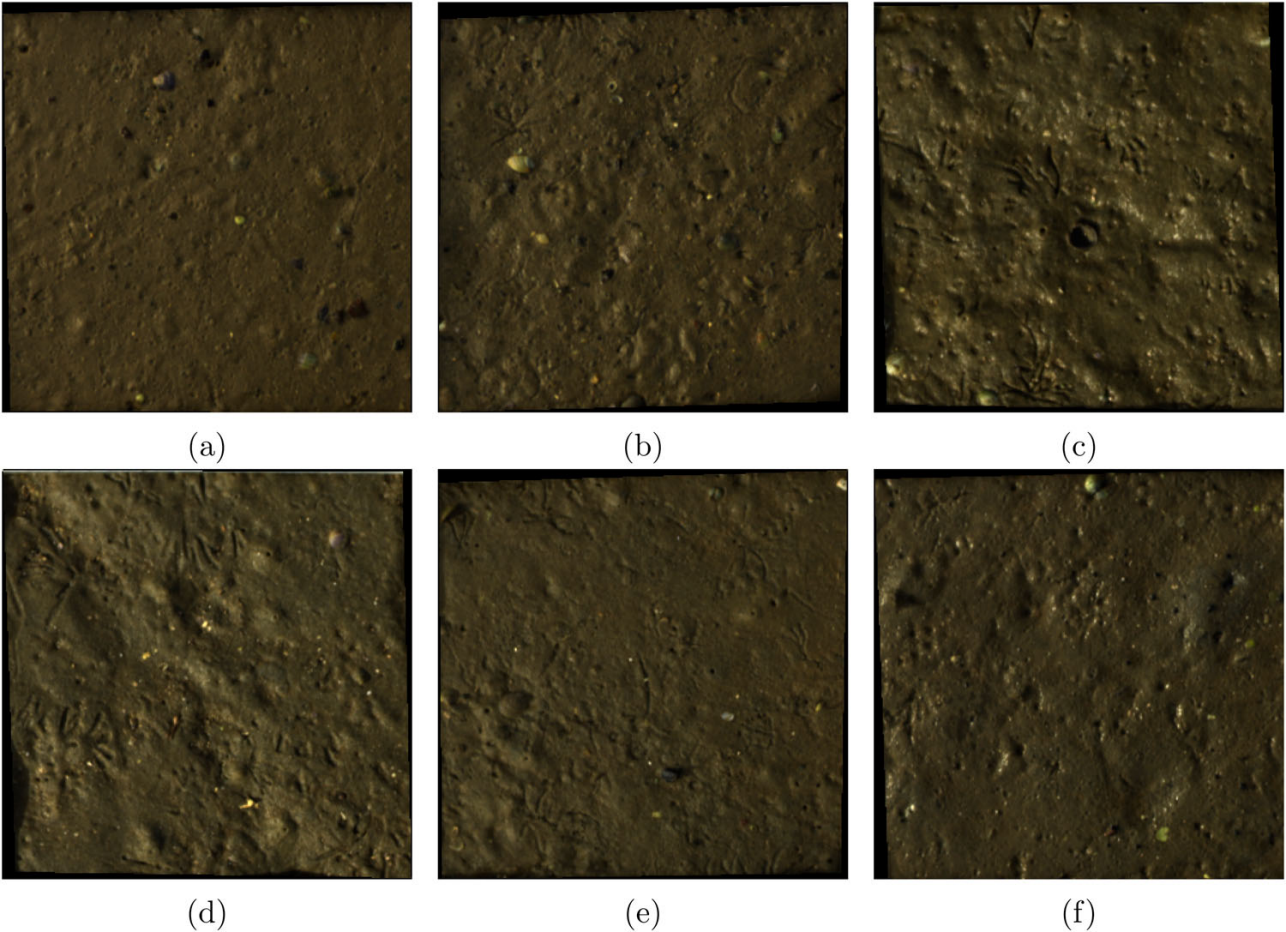
RGB images of full quadrats containing the sub-regions with the highest, lowest and closest to the median porosity (top row), and organic matter (bottom row) values. The porosity values correspond to **A,** 30.969, **B,** 39.934, and **C,** 54.609, and organic matter content values of **D,** 0.518, **E,** 1.051, and **F,** 2.091.

The spectral signatures of each quadrat were averaged into a single mean reflectance spectra per quadrat and are plotted in Figure 5. Two quadrat subregions were observed to have a higher average reflectance owing to water saturation, causing a higher reflectance on the surface of the sediment despite specular regions being segmented out. The SNV preprocessing technique corrects this by standardising the reflectance curves. The atmospheric absorption of $H_{2}O$ between 920 and 970 nm and intermittent cloud cover cause high variations in the spectra in the NIR range (Behmann et al. 2018). By visually inspecting the full quadrats containing the highest and lowest porosity and organic matter values (Figure 6), it is difficult to see the differences between the sediment areas. The lowest porosity values were found in the final quadrat, which was in a sandy area further inshore than the other quadrats. Based on these examples, it appears that porosity increases with sediment saturation, as the example with the highest porosity contains more specular highlights, indicating surface-level moisture.

### Regression models

We first fitted the regression models to predict the attributes of interest and benchmark against the wavelength selection methods. Table 5 lists the $R^{2}$ and MSE values for the models trained to predict the porosity and organic matter content of the calibration set using five-fold cross-validation. Cross-validation was performed on the training set, with the training metrics representing the average performance of the models on the four training folds, and the testing metrics representing the average performance of the models on the fifth fold. The $R^{2}$ and MSE values of the models applied to the test set were determined by averaging the performance of the models trained on four training folds applied to the full unseen test set.

**Table 5.** Sediment porosity and organic matter content prediction based on the full wavelength set using SVR and PLSR.

Attribute	Model	Training set				Test set	
		Training				Testing	
		$R_{c}^{2}$	$\mathrm{MS}E_{c}$	$R_{\mathrm{cv}}^{2}$	$\mathrm{MS}E_{\mathrm{cv}}$	$R_{p}^{2}$	$\mathrm{MS}E_{p}$
Porosity	PLSR (LVs=8)	0.4356	9.1376	−0.1898	15.9551	0.2547	4.1304
	SVR-RBF	0.2106	12.7886	−0.0539	15.5982	0.2305	4.2642
	SVR-Linear	0.3758	10.1004	**−0.0488**	**15.2081**	**0.3434**	**3.6388**
	XGBoost	0.9908	0.1524	−0.1036	15.4024	0.2613	4.0934
	LightGBM	0.9718	0.4615	−0.221	16.3157	0.2797	3.9919
Organic matter	PLSR (LVs=10)	0.7706	0.0276	0.5213	0.0513	**0.506**	**0.0371**
	SVR-RBF	0.8581	0.0171	0.5073	0.0575	**0.5013**	**0.0374**
	SVR-Linear	0.908	0.0111	**0.5535**	**0.0511**	0.3951	0.0454
	XGBoost	0.8616	0.0168	0.2575	0.0849	0.2419	0.0569
	LightGBM	0.8194	0.0218	0.3065	0.0806	0.2401	0.0571

Across both attributes, there was a significant drop in the predictive performance between the training and testing portions of the cross-validation. The cross-validation $R_{\mathrm{cv}}^{2}$, which is the $R^{2}$ performance on the hold-out fold averaged over the five folds, is negative for porosity. Negative $R^{2}$ values indicate that the sum of the squared differences between the predictions and ground-truth values is greater than the sum of the squared differences between the attribute mean and ground-truth values.

The drop in performance between the average calibration model performance and the prediction set performance suggests that these methods are finding trends in the data that do not generalise well to unseen data and may be limited by the small amount of available data or the spectral range captured. These full-wavelength models poorly predict sediment porosity; therefore, porosity was not considered in further wavelength selection experiments. There was no clear best model, with the PLSR and SVR-RBF models performing almost equally in terms of the cross-validation and test metrics for organic matter, and the SVR-linear model performed better in predicting porosity.

The XGBoost and LightGBM models overfitted to the training folds worse than other standard models. A simple grid search was insufficient to optimise the parameters due to the small dataset size. The parameters were manually tuned to minimise overfitting. These two models were not considered in combination with the wavelength selection models in the following sections because of their poor performance.

### Wavelength selection

To evaluate the performance of the various wavelength selection techniques, we first examine the performance of the GP method over generations and then compare the performance between the methods.

#### GP method performance across generations

**Figure 7.**
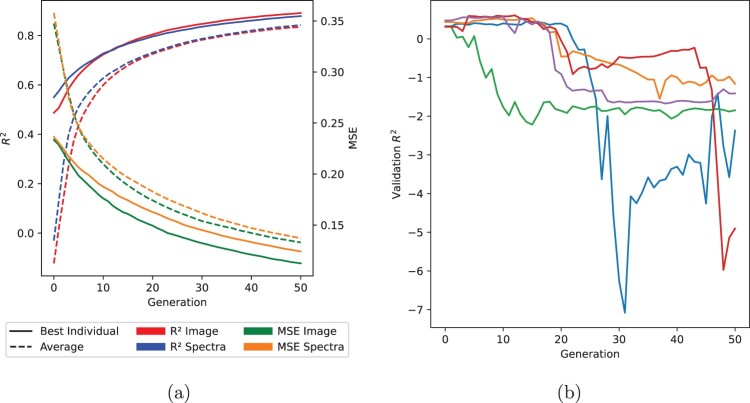
**A,** Average and best fitness performances of the various implementations averaged over the 50 runs. **B,** Five examples, each coloured differently, of anomaly runs where validation performance dropped below $R^{2}$ of −1.

Figure 7A shows the best and average fitness performance of the spectra- and image-based methods using both fitness functions throughout the evolutionary process, averaged (median) over the 50 runs. Throughout the evolutionary process, the fitness values of the populations improve initially at a high rate, with diminishing improvements as generations increase. The average gap between the best individuals from the two MSE implementations appears to widen throughout the evolutionary process, which may indicate that the image-based method performed better. Between the two $R^{2}$ implementations, there is only a small difference in the average and fitness performance, but comparatively, the image-based MSE implementation appears to outperform the spectra-based method. This may indicate that the MSE fitness function provides a better error surface for population optimisation. Nonetheless, this graph does not allow us to determine whether one fitness function is superior to another.

One observation from the GP implementations was a rare drop in the validation performance during the evolutionary process. Of the 50 runs for each fitness function, this was observed in three runs for the spectra-based implementation using the MSE fitness function, once for the spectra-based implementation using the $R^{2}$ fitness function, and once for the image-based implementation using the $R^{2}$ fitness function. These runs are plotted in Figure 7B. In each of these runs, a previously unseen individual that further maximises (in the case of $R^{2}$ as a fitness function) or minimises (for MSE) the fitness function entered the population through mutation or crossover. For spectra-based implementations, this change is equivalent to selecting new wavelengths or removing previously selected ones. However, in these cases, the new predictive relationship found by the SVR model trained on the full calibration set did not generalise well to the unseen prediction set.

**Figure 8.**
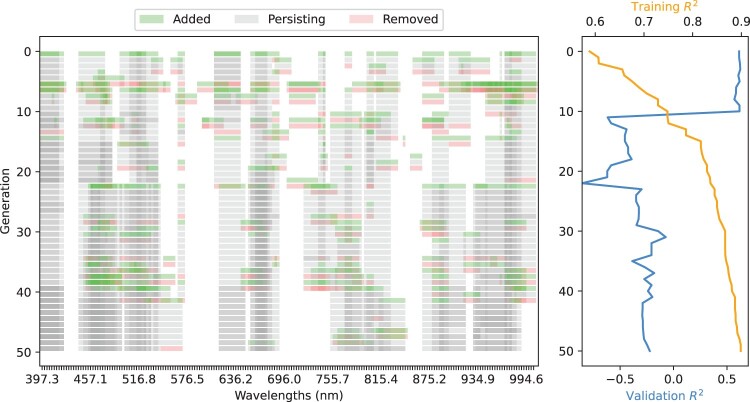
Spectral regions selected by one anomalous run of the spectra-based GP method. The wavelengths selected by the best individual of each generation are illustrated by the graph on the left, with shading indicating whether a spectral feature was added (green) or removed (red) between generations. The graph on the right plots the fitness value of the best individual over generations.

Figure 8 shows the best individual across generations to visualise the changes in the selected features and their effect on the fitness function performance and test $R^{2}$. One anomalous run was selected from the spectra-based $R^{2}$ runs, which exhibited this rare drop in performance. In the top example, the first drop in the test $R^{2}$ value from 0.62 to −0.62 occurs when, among other wavelengths, an interval centred at 809.5 nm is added in the 10th generation. After this generation, this wavelength interval remains in the set selected by the best individual for the remaining generations. Other wavelengths were added and removed; however, no change in the performance was observed. This indicates that this may be a noisy feature correlated with the ground truth for the training set; however, this relationship does not generalise to unseen data.

In another example, a drop in performance from a test $R^{2}$ of 0.525 to −3.4628 occurs in the 15th generation, and in the following generation, this rebounds to an $R^{2}$ of 0.599. Investigating the wavelengths utilised by the best individuals as the performance drops, a wavelength interval centred at 809 nm is present again in the individual with the highest fitness from Generation 15 and not in the set extracted by the best individuals from Generation 14 or 16. The individuals in this run evolved to include a larger feature set through the evolutionary pressure, which led to a denser graph which makes it difficult to determine which wavelength inclusion or exclusion caused this drop. This investigation suggests that these GP models may find noisy correlations between features that do not generalise well to unseen data in this dataset as well as potentially in other real-world datasets. This may be a limitation of having a small dataset and the randomness of the GP methods. Given a different dataset split, the comparison methods may experience similar differences in performance between the calibration and prediction sets.

#### Performance of wavelength selection techniques

For each comparison wavelength selection method, PLSR and SVR (RBF and linear kernels) models were fitted. Linear SVR models were fitted for the GP methods because these were the models the feature set was optimised for during the evolutionary process. PLSR models were fitted to show that the extracted features can be utilised with other methods after the evolutionary process. Each wavelength selection model was trained five times using cross-validation on the calibration set selected by the SPXY dataset splitting algorithm, and the evaluation performance is presented in Table 6. The training (calibration) metrics ($R_{c}^{2}$ and $\mathrm{MS}E_{c}$) represent the average performance of the model on the training portion of the five-fold cross-validation, whereas the cross-validation metrics ($R_{\mathrm{cv}}^{2}$ and $\mathrm{MS}E_{\mathrm{cv}}$) indicate the average performance on the testing portion of the five-fold cross-validation. The prediction metrics ($R_{p}^{2}$ and $\mathrm{MS}E_{p}$) indicate the average performance of the models trained on four folds of the calibration set and applied to the entire test (prediction) set. The number of wavelengths selected by each method is recorded in column *n* with the average and standard deviation recorded for the GP runs.

**Table 6.** Sediment organic matter content prediction based on wavelength selection models. For the GP models the mean number of unique wavelength/width pairs selected, and the median values for the performance metrics ($R^{2}$ and MSE) are presented with the standard deviation of the values in parentheses. The best results have been bolded.

Model	*n*	Training set				Test set	
		Training		Testing			
		$R_{c}^{2}$	$\mathrm{MS}E_{c}$	$R_{\mathrm{cv}}^{2}$	$\mathrm{MS}E_{\mathrm{cv}}$	$R_{p}^{2}$	$\mathrm{MS}E_{p}$
CARS-PLSR (LVs=18)	26	0.9062	0.0113	0.7522	0.0282	0.3959	0.0454
CARS-SVR-RBF	26	0.8883	0.0135	0.7155	0.0325	0.4139	0.044
CARS-SVR-Linear	26	0.888	0.0135	**0.7934**	**0.0235**	0.4899	0.0383
SPA-PLSR (LVs=17)	11	0.5555	0.0537	0.3763	0.0709	0.3472	0.049
SPA-SVR-RBF	11	0.6926	0.0372	0.4697	0.062	0.3507	0.0488
SPA-SVR-Linear	11	0.5275	0.0571	0.4042	0.0686	0.2973	0.0528
RC-PLSR (LVs=20)	22	0.8024	0.0238	0.5284	0.0531	0.3439	0.0493
RC-SVR-RBF	22	0.8239	0.0212	0.5616	0.0505	0.44	0.0421
RC-SVR-Linear	22	0.7234	0.0333	0.5426	0.0507	0.4891	0.0384
GP-Spectra-$R^{2}$-SVR	41.34 (17.78)	0.7998 (0.0918)	0.0242 (0.0111)	0.4694 (0.3139)	0.0398 (0.0236)	0.614 (0.3601)	0.043 (0.0267)
GP-Spectra-$R^{2}$-PLSR		0.827 (0.1059)	0.0209 (0.0128)	0.3315 (0.323)	0.0502 (0.0243)	0.6011 (0.3327)	0.0441 (0.0266)
GP-Spectra-MSE-SVR	47.3 (20.24)	0.829 (0.08)	0.0206 (0.0097)	0.4511 (0.7039)	0.0412 (0.0529)	**0.6527** (0.0758)	**0.0393** (0.0091)
GP-Spectra-MSE-PLSR		0.8449 (0.0671)	0.0187 (0.0081)	0.3603 (0.5109)	0.048 (0.0384)	0.644 (0.0654)	0.0396 (0.0074)
GP-Image-$R^{2}$-SVR	34.34 (11.84)	0.7416 (0.0824)	0.0311 (0.01)	0.2809 (0.5267)	0.054 (0.0396)	0.5135 (0.1064)	0.0547 (0.0114)
GP-Image-$R^{2}$-PLSR		0.7668 (0.0845)	0.0281 (0.0102)	0.2599 (10.0539)	0.0556 (0.755)	0.4919 (0.099)	0.0554 (0.011)
GP-Image-MSE-SVR	28.58 (11.73)	0.6949 (0.0966)	0.0368 (0.0117)	0.29 (0.1565)	0.0533 (0.0117)	0.4972 (0.1391)	0.0567 (0.0168)
GP-Image-MSE-PLSR		0.7271 (0.0957)	0.033 (0.0116)	0.2479 (0.1403)	0.0565 (0.0105)	0.4915 (0.1155)	0.0569 (0.0133)

A comparison of the GP-based methods to the other wavelength selection methods shows all GP-based methods outperformed the other methods on the prediction set. As shown in the test performance graphs in Figure 7B, a few GP runs had extremely poor results when tested using cross-validation, resulting in large negative $R^{2}$ values that skewed the averages and led to larger standard deviations results. To account for this, the results presented in Table 6 are the median values over 50 runs.

The wavelengths selected by the CARS method, when used for regression using PLSR and linear SVR models, showed good performance on the calibration and cross-validation folds; however, the performance decreased when applied to the unseen prediction set. It is worth noting that the CARS method selects a subset of wavelengths using the RMSE from cross-validation, which could mean that the CARS method over-optimises the cross-validation performance. The linear SVR model fitted on the wavelength subset selected by the SPA exhibited the lowest performance on the prediction set out of the comparison methods, with an $R_{p}^{2}$ value of 0.2973. The SPA method first selects subsets of wavelengths based on projections to minimise collinearity, and the final subset is selected based on the cross-validation performance. This implies that informative wavelengths may be screened out in the first stage based on collinearity since model performance is not taken into consideration.

The image-based methods performed worse than the spectra-based models across all of the performance metrics. Although the image-based program structure can represent the same feature extractors as the spectra-based program structure by omitting the image features, the larger feature space likely leads to a more difficult search for optimal individuals. The larger search space may have also provided the image-based models with more noisy features that could lead to overfitting. Across all three sets, the spectra-based GP method had a lower MSE and a higher $R^{2}$. The best individuals from the spectra-based models had $R_{p}^{2}$ values of 0.73 and 0.75 for the $R^{2}$ and MSE fitness functions, respectively. Similarly, the best image-based individuals achieved $R_{p}^{2}$ values of 0.66 and 0.65 for the $R^{2}$ and MSE fitness functions, respectively.

There was only a slight difference between the linear SVR and PLSR models applied to the evolved GP trees; the PLSR models generally fitted better to the training folds and performed slightly worse in terms of cross-validation and testing metrics. No noticable difference was found between the two fitness functions. Some results from the GP methods had a high standard deviation caused by some anomalous runs discussed previously, which did not generalise well to the test sets, leading to large negative $R^{2}$ values. However, the majority of the GP runs outperformed the comparison methods. A fitness function that captures the generalisation performance better in lieu of a larger dataset may resolve this problem.

### Selected wavelengths across methods

The SPA, CARS, and RC methods selected 11, 26, and 22 wavelengths, respectively. The number of wavelengths selected for each method was reasonably high, which may indicate that there were no clear indicators of organic matter in the 397–1004 nm spectral range. The number of wavelengths selected on average for the top spectra-based GP individuals varied across the 50 runs but averaged 41.34 unique wavelengths with a minimum and maximum of 13 and 109 for the individuals evolved using the $R^{2}$ fitness function, and an average of 47.3 unique wavelengths with a minimum and maximum of 17 and 120, respectively, for the individuals evolved using the MSE fitness function. A unique wavelength range can be defined as a unique pair of centre wavelengths and widths.

The image-based methods selected fewer wavelengths, with the MSE fitness function selecting an average of 28.58 unique wavelengths, with a minimum and maximum of 11 and 60, respectively, and the $R^{2}$ fitness function individuals selecting an average of 34.34 unique wavelengths, with a minimum and maximum of 11 and 65, respectively. The number of feature extraction branches, which could include duplicate wavelengths with different processing applied, was also higher with spectra-based individuals. The difference between the image-based and spectra-based implementations can be attributed to the difference in the tree size constraints and the larger number of data-type transitions required to transform a hyperspectral image into a wavelength feature. These constraints were put in place to limit the computational time, as image-based populations take significantly longer to evaluate.

**Figure 9.**
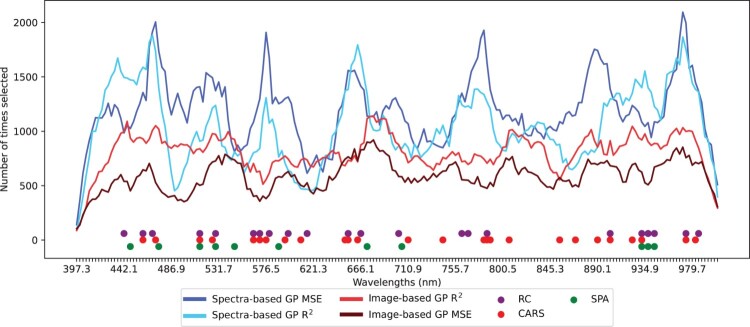
Number of times each wavelength was selected by the four GP implementations (Spectra- and image-based with $R^{2}$ and MSE fitness functions) across the top 10 individuals over 50 random runs compared to other wavelength selection algorithms.

There were also clear regions where spectral information was less useful, such as 500 nm, 600–650 nm and 800–890 nm, each of which corresponded to troughs in the number of times each wavelength was chosen by the GP methods and less frequent selection of wavelengths by the three comparison methods. Figure 9 plots the number of times each wavelength was selected by the two spectra-based and two image-based GP methods across the top 10 individuals over each of the 50 runs, and the wavelengths selected from the comparison methods. Minimal differences were observed in the common wavelength regions between the two spectra-based fitness functions and image-based implementations. One of the differences was between the two image-based methods, where the implementation based on the $R^{2}$ fitness function selected more features in the 440–540 nm range than the MSE implementation. The difference between the peaks and troughs was more pronounced for the spectra-based methods, with the MSE individuals being 3.41 times more likely to select a wavelength feature centred at 968 nm than one centred at 614 nm. Both spectra-based methods were between 2.53 and 2.66 times more likely to select a feature centred on 678 nm than 409 nm.

Despite the similarities in wavelength selections from individuals sharing the same program structure, the peaks of the spectra-based selections did not correspond to those of the image-based selections. Examples include the range of 750–800 nm, where there are prominent peaks for the spectra-based methods and wavelength selections from the RC and CARS methods, but no increase for the image-based methods, and similarly for 550–620 nm. Furthermore, the image-based implementations had lower variations between the peaks and troughs, suggesting that wavelengths with informative image features were not necessarily the same as those with informative spectral features.

### Analysis of selected functions

To compare the effectiveness of the individual functions within the function sets, the frequency of each function was compared with that of other functions that shared the same return type. Frequency was measured by counting the occurrences across the top 10 individuals in each run for image-based and spectra-based implementations. Functions were compared across functions that shared the same input and return types to avoid a bias towards functions closer to the leaves and functions that do not change the feature type and could be included more than once within a branch. Therefore, this frequency measurement comparison indicates the functions that are more effective in their particular roles.

First, analysing the image-based implementation, the spectral extraction function was the most common operation in the first stage (39% of operations), followed by the Gaussian and mean interval image selection functions, which were selected approximately 30% of the time. The median image interval selection method is infrequently found among the best individuals, at less than 1%. Spectral preprocessing functions (SNV and Savitzky–Golay filtering) were applied to approximately 10% of the features extracted from the spectra in the images, which may indicate that they are not as important for this application. Most importantly, the image features, such as the five GLCM features and the statistical features, were largely uninformative, with 74% of the image features coming from a simple average and 5% from the standard deviation. From the spectra processing functions, there was very little difference between the mean and median interval selection functions across both implementations, with the median selection function being the most common. Across both implementations, larger feature concatenation functions were favoured, allowing the GP tree to include more features.

### Application of organic matter model

The best GP individual from the 50 runs of the spectra-based implementation using the MSE fitness function was chosen to predict the organic matter content of all 750 subregions in the 30 quadrats. A linear SVR model based on the features extracted by the GP model was fitted using all training set subregions with corresponding ground-truth measurements. The 750 subregions included 600 subregions for which the organic matter content was not measured because of the cost and time requirements to do so. The spectra-based GP implementations showed better performance than the image-based implementations and were simpler to interpret; hence, the best spectra-based individual was selected. The selected individual achieved an $R_{p}^{2}$ of 0.58, and performed well in terms of $R_{\mathrm{cv}}^{2}$ with a score of 0.63.

**Figure 10.**
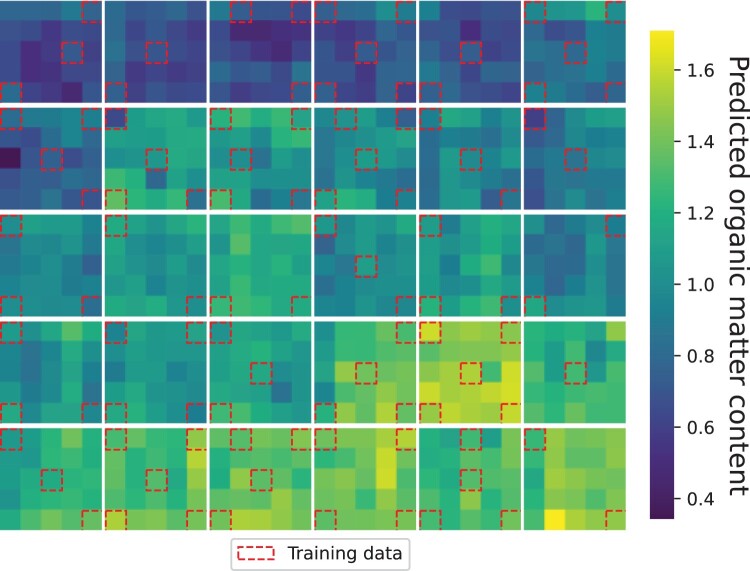
Prediction of organic matter content across the 30 quadrat plots by the best GP individual. The samples used for training are marked with a red rectangle.

Figure 10 shows the predictions of organic matter content with the samples used for fitting the SVR model, delimited by a red square. In the majority of quadrats, there was very little variation between subregions, with more variation between the quadrats. 23 wavelength ranges were selected by the GP tree of this individual, with many ranges selected multiple times, such as four around 940 nm and two around 576 nm. This individual utilised 18 unique wavelength centre/width pairs, which is fewer than all comparison wavelength selection methods. The number of wavelengths could be further reduced through manual pruning by determining which wavelengths had the least impact on the model prediction, or by substituting previously selected wavelengths for wavelengths with similar ranges.

## Conclusion

This study collected hyperspectral images of sediment from an intertidal area, and porosity and organic matter content were measured across 30 sampling quadrats. Two flexible GP-based approaches were proposed and compared, integrating a typical hyperspectral imaging pipeline's wavelength selection and feature extraction steps using two fitness functions ($R^{2}$ and MSE). Standard regression models (SVR, PLSR, XGBoost, and LightGBM) were used to predict the organic matter content and porosity. SVR and PLSR models were fitted to predict the organic matter content with reasonable accuracy; however, these models could not reliably predict porosity, which was ignored in further analyses.

The GP individuals evolved using both implementations performed well across the calibration, cross-validation, and prediction metrics compared to three common wavelength selection methods, SPA, CARS, and RC, while still reducing the required wavelengths. No noticeable differences were observed between the two fitness functions; however, the spectra-based GP methods performed better than the image-based implementations, indicating that image features may not be as informative for this task or that a larger search space makes finding better solutions more challenging. This was supported by the texture features being less commonly utilised than the spectral features in the best-evolved GP function trees. Given a different application where the texture features are more informative, the image-based model may perform better. The features extracted by the GP trees were found to be usable by similar regression models without a drop in performance.

A few evolutionary runs of the GP methods exhibited a significant drop in performance between the training (i.e. fitness value) and the testing performance. Investigating the features extracted by two of these runs revealed that one particular wavelength interval may have influenced the generalisation ability of the subsequent SVR models.

Finally, one of the best spectra-based individuals was applied to predict the organic matter content of all subregions across the 30 quadrats. This particular model achieved an $R_{p}^{2}$ value of 0.625, and when applied to all subregions, the predictions showed low variability within each quadrat, with a higher variation between the quadrants. This demonstrates the applicability of hyperspectral imaging and wavelength selection for rapid assessment of benthic habitat health.

Overall, the proposed GP-based methods show promise as more flexible wavelength selection alternatives. Compared with other wavelength selection methods, the proposed method allows for variable-sized wavelength intervals and can incorporate various feature types, such as texture features. This allows users to evolve more complex feature extraction trees that are still interpretable without extensive expert knowledge. Future work may expand on this method by using data augmentation, transfer learning, or other few-shot learning techniques to improve the performance of this model on small datasets such as this one.

## Data Availability

The dataset collected and used in this study is openly available in FigShare at 10.17608/k6.auckland.25546396.

## Note

1 https://github.com/mrog173/Hyperspectral-Regression-GP.
